# A Novel Variational Bayesian Method with Unknown Noise for Underwater INS/DVL/USBL Localization

**DOI:** 10.3390/s25123708

**Published:** 2025-06-13

**Authors:** Haoqian Huang, Chenhui Dong, Yutong Zhang, Shuang Zhang

**Affiliations:** College of Artificial Intelligence and Automation, Hohai University, Changzhou 213200, China; chenhui_dong@hhu.edu.cn (C.D.); yutongz@hhu.edu.cn (Y.Z.); zhangshuang98@hhu.edu.cn (S.Z.)

**Keywords:** Kalman filter, inverse-Wishart distribution, non-Gaussian noise, variational Bayesian, underwater vehicle

## Abstract

In the complex underwater environment, it is hard to obtain accurate system noise prior information. If uncertainty system noise model is used in state determination, the precision will decrease. To address the problem, this paper proposes a novel inverse-Wishart (IW) based variational Bayesian adaptive cubature Kalman filter (IW-VACKF), and the inverse-Wishart distribution is employed as the conjugate prior distribution of system noise covariance matrices. To improve the modeling accuracy, a mixing probability vector is introduced based on the inverse-Wishart distribution to better characterize the uncertainty and dynamic of state noise in underwater environments. Then, the state transition and the measurement process are derived as hierarchical Gaussian models. Subsequently, the posterior information of the system is jointly calculated by employing the variational Bayesian method. Simulations and real trials illustrate that the proposed IW-VACKF can improve the state estimation precision efficiently in the complex underwater environment.

## 1. Introduction

**Figure 1:**
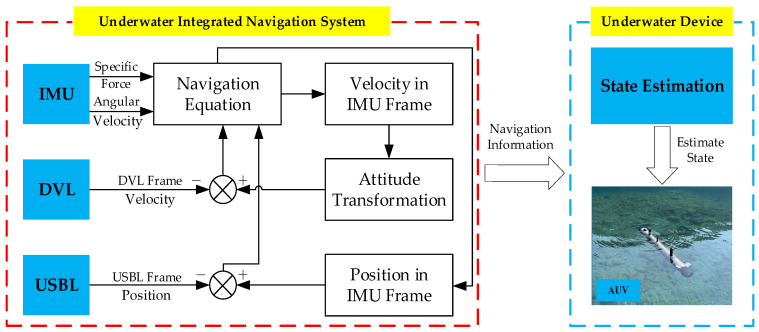
Underwater navigation system.

With the rapid development of ocean exploration, underwater navigation technology has attracted increasing attention across various fields [1,2]. Underwater environments present unique challenges for signal transmission, as electromagnetic signals cannot propagate effectively over long distances. In contrast, acoustic signals exhibit superior propagation capabilities, making acoustic-based sensors indispensable for underwater positioning [3,4,5]. Figure 1 illustrates the structure of a typical underwater navigation system, which relies on the integration of multiple sensors, including Doppler Velocity Log (DVL), Inertial Measurement Unit (IMU), and Ultra-Short Baseline (USBL). Each sensor plays a critical role in providing essential navigation data. The DVL and USBL utilize acoustic signals to measure the velocity and position of underwater devices [6,7,8], while the IMU calculates position and velocity based on angular velocity and specific force measurements [9]. By fusing data from these sensors, the system can provide accurate and reliable navigation information [10,11]. However, the performance of these sensors is significantly affected by the complex and noisy underwater environment. Acoustic signals are susceptible to interference, leading to inaccuracies in the measurements provided by DVL and USBL. Additionally, the data of IMU can drift over time due to the accumulation of errors. These challenges highlight the need for robust data processing algorithms to mitigate the impact of noise and ensure the reliability of the navigation system [12,13]. Therefore, the accuracy and reliability of underwater navigation systems largely depend on the ability to address the challenges associated with different sensors.

To address these problems, data fusion techniques based on state estimation theory have been widely adopted. The extended Kalman filter (EKF) is a powerful technique for position estimation, which is derived based on the Taylor’s formula and Bayes principle. However, when the system is strongly nonlinear, the EKF usually performs poorly. With the application of the unscented transformation, the unscented Kalman filter (UKF) has a satisfactory accuracy in a system with extreme nonlinearity. Unfortunately, the UKF suffers from the curse of dimensionality. When the system is a high-dimensional state space, the accuracy of UKF will decrease significantly. By introducing the cubature sampling rule, the cubature Kalman filter (CKF) avoids the curse of dimensionality [14,15,16,17].

However, the above algorithms are fundamentally limited by their assumption of precisely known noise statistics, a requirement rarely met in practical underwater environments. The prior information of the true noise covariance matrix (TNCM) plays an essential role in the state estimation of the above algorithms. An incorrect noise covariance matrix (NCM) may cause the filter to produce significant determination errors or even experience filtering divergence [18,19]. In the underwater environment, due to the unknown disturbance, the TNCM is hard to determine and may be time-varying. To specifically address these challenges posed by unknown noise characteristics in the underwater localization, various adaptive Kalman filters (AKF) have been investigated. For instance, by reconstructing the model and introducing the maximum information potential (MIP) criterion with adaptive kernel bandwidth, a novel adaptive filtering algorithm to address compass failure and non-Gaussian measurement noise in multi-AUV cooperative localization is proposed [20]. Using variational Bayesian (VB) to estimate measurement noise covariance and a variable kernel width MCC strategy to suppress outliers, a VB-based Maximum Correntropy Criterion (MCC) adaptive Kalman filter can tackle unknown, time-varying noise and measurement outliers in SINS/USBL integrated navigation systems [21]. Furthermore, using VB inference for state and process and measurement noise covariances (PMNC) estimation, a variational Bayesian adaptive Kalman filter for scenarios with inaccurate PMNC and outliers, modeling state transition and measurement likelihood state probability density function (PDF) as Gaussian–Gamma mixture distributions [22]. These studies represent significant efforts to improve positioning accuracy by more effectively modeling and mitigating the impact of complex underwater noise and system uncertainties.

These AKFs are usually considered an effective approach to address the issue. The AKF obtains the optimal state estimation by adaptively adjusting the filtering parameters based on system measurement and working environment [23]. The VB-based AKF (VBAKF) is a classical adaptive Kalman filtering algorithm. To achieve precise state estimation, the VBAKF models the state transition and measurement process by selecting an appropriate conjugate prior distribution; thereby, the joint PDF is established. Then, the system state can be approximately acquired by minimizing the Kullback–Leibler divergence (KLD) between the prior and posterior PDF [24,25]. But the VBAKF cannot provide accurate state estimation in nonlinear systems. To solve this problem, the VB method is usually combined with linear approximation approaches, such as unscented transformation or the cubature sampling rule, to perform state determination in nonlinear system. The robust CKF based on VB and transformed posterior sigma points error and the inverse-Wishart-based VBCKF (VBCKF-QR) are all designed based on this method [26]. In the underwater environment, due to various unknown disturbances underwater such as ocean current impact and electromagnetic interference, the state noise is often significantly more complex than measurement noise.

By introducing a mixing probability vector to describe the statistical characteristics of state noise, the Gaussian-inverse-Wishart mixture (GIWM) distribution is designed. By introducing the mixing probability vector into the modeling of system noise, the GIWM is suitable for modeling this complex noise with a strongly inaccurate covariance matrix. By utilizing the VB approach to estimate system state, the variational AKF with the GIWM distribution (VAKF-GIWM) is proposed [27]. Nevertheless, the VAKF-GIWM has two disadvantages: Firstly, it is hard to acquire the analytical solution of system state in a non-linear system. Secondly, in the process of modeling state transition, it is necessary to use the previous state, leading to the repeated estimation of the state at the last time, so the application scope of the VAKF-GIWM is limited due these problems.

In order to accurately estimate the system state in underwater nonlinear system, a novel inverse-Wishart based variational Bayesian adaptive cubature Kalman filter (IW-VACKF) is proposed in this paper. By introducing the mixing probability, the state transition process is modeled and the cubature sampling rule is integrated into the approximate calculation of the nonlinear integral part. Meanwhile, Taylor’s formula is utilized to simplify the nonlinear calculate part in the variational iterative process. To avoid repeated calculations of the system state from the previous step, the state estimation results from the last moment are directly used in the iterative calculation at the current moment. The highlights of this work are shown below:

(1) To accurately describe underwater noise, the IW distribution and mixing probability vectors are introduced, and the state transition and the measurement process are derived as hierarchical Gaussian models.
(2) To acquire the optimal position estimation, the IW-VACKF is proposed by using the VB method and cubature sampling rule.
(3) The proposed IW-VACKF has been successfully validated in practical experiments, demonstrating superior performance in enhancing the accuracy and robustness of multi-sensor integrated navigation systems under complex underwater environments.

The main structure of this paper is formed below. Section 2 shows that the formulation of the nonlinear state-space model is displayed and the formulation of the cubature sampling rule is presented. In Section 3, the state transition and system measurement are described by inverse-Wishart distribution and the mixing probability vector. Section 4 introduces the IW-VACKF. In Section 5, the proposed IW-VACKF is compared with the UKF, CKF, VBCKF-QR, and the optimal filter, under simulations and surface vessel trials performed in the Yangtze River, China. Finally, the conclusion is drawn in Section 6.

## 2. Problems Model and Cubature Kalman Filter

### 2.1. State-Space Model for INS/DVL/USBL Integrated Navigation

To achieve accurate and reliable navigation information, the problem model considered in this paper is shown:

(1) $$\left\{\begin{array}{l} x_{k}=f\left(x_{k-1}\right)+w_{k-1}\\ z_{k}=h\left(x_{k}\right)+v_{k} \end{array}\right.$$

where $x_{k}\in {\mathbb{R}}^{m}$ and n represent the state vector and the dimension of the state vector, and $x_{k}$ include velocity state vector and position state vector; $z_{k}\in {\mathbb{R}}^{m}$ and m are the measurement vector and the dimension of the measurement vector, and $z_{k}$ include measurement data at the time k for USBL, DVL, and IMU; $f\left(\cdot \right)$ and $h\left(\cdot \right)$ represent the state transition function and the measurement update function, usually non-linear; $w_{k-1}$ and $v_{k}$ are the system state noise in time k−1 and measurement noise in time k, respectively; and they are fit the Gaussian distribution, i.e., $w_{k-1}\sim N\left(0,Q_{k-1}\right)$ and $v_{k}\sim N\left(0,R_{k}\right)$ [28]. However, in the underwater environment, the NCM and $R_{k}$ are time-varying and non-constant; the system of Equation (1) is called as non-Gaussian noise system.

### 2.2. Cubature Kalman Filter

According to the model (1) and cubature sampling rule principle [29], the prior PDF $p\left(x_{k}\right)$ is modeled by $x_{k-1}\sim N\left({\hat{x}}_{k|k-1},P_{k|k-1}\right)$. ${\hat{x}}_{k|k-1}$ is the one-step prediction of state vector; $P_{k|k-1}$ the one-step prediction error covariance matrix; $x_{0}$ is the initial state vector, and it satisfies $N\left({\hat{x}}_{0|0},P_{0|0}\right)$ which is independent with state noise $w_{k-1}$ and measurement noise $v_{k}$. Based on the above conditions, the posterior PDF $p\left(x_{k}\right)$ at time *k* can be determined with the posterior PDF $p\left(x_{k-1}\right)$ and the measurement $z_{k}$. Suppose $x_{k}\sim N\left(\hat{x},P_{k|k}\right)$, and $\xi_{i}$ for CKF is selected as:

(2) $$\xi_{i}=\left\{\begin{array}{l} \sqrt{n}e_{i},\;\;i=1,2,\ldots ,n\\ -\sqrt{n}e_{i-n},\;\;i=n+1,n+2,\ldots ,2n \end{array}\right.$$

where $e_{i}$ is the *i*-th elementary column vector. Define root mean square $S_{k|k-1}=\mathrm{chol}\left(P_{k|k-1}\right)$, $S_{k-1|k-1}=\mathrm{chol}\left(P_{k-1|k-1}\right)$ and *m* = 2*n*. The mean state vector ${\hat{x}}_{k|k}$ and error covariance matrix $P_{k|k}$ can be determined by employing the CKF as:

(1) Time update

By using (3), the cubature points $X_{i,k-1|k-1}^{+}$ are updated:

(3) $$X_{i,k-1|k-1}^{+}=S_{k-1|k-1}\xi_{i}+{\hat{x}}_{k-1|k-1},\;S_{k-1|k-1}=\mathrm{chol}\left(P_{k-1|k-1}\right)$$

where i=1,…,m.

Determine the state predicted vector and covariance predicted matrix as follows:

(4) $$\left\{\begin{array}{l} {\hat{x}}_{k|k-1}=\sum_{i=1}^{m}\omega_{i}f\left(X_{i,k-1|k-1}^{+}\right)\\ P_{k|k-1}=\sum_{i=1}^{m}\omega_{i}f\left(X_{i,k-1|k-1}^{+}\right)f{\left(X_{i,k-1|k-1}^{+}\right)}^{Τ}-{\hat{x}}_{k|k-1}{\hat{x}}_{k|k-1}+Q_{k-1} \end{array}\right.$$

(2) Measurement update

Renew cubature points as:

(5) $$X_{i,k|k-1}^{-}=S_{k|k-1}\xi_{i}+{\hat{x}}_{k|k-1},\;S_{k|k-1}=\mathrm{chol}\left(P_{k|k-1}\right)$$

Calculate measurement predicted vector and covariance predicted matrices as:

(6) $$\left\{\begin{array}{l} {\hat{z}}_{k|k-1}=\sum_{i=1}^{m}\omega_{i}h\left(X_{i,k|k-1}^{-}\right)\\ P_{zz,k|k-1}=\sum_{i=1}^{m}\omega_{i}h\left(X_{i,k|k-1}^{-}\right)h{\left(X_{i,k|k-1}^{-}\right)}^{Τ}-{\hat{z}}_{k|k}{\hat{z}}_{k|k-1}^{Τ}+R_{k}\\ P_{xz,k|k-1}=\sum_{i=1}^{m}\omega_{i}X_{i,k|k-1}^{-}h{\left(X_{i,k|k-1}^{-}\right)}^{Τ}-{\hat{x}}_{k|k}{\hat{z}}_{k|k-1}^{Τ} \end{array}\right.$$

Then the system state vector ${\hat{x}}_{k|k}$ and the predictive error covariance matrix $P_{k|k}$ can be estimated as:

(7) $$\left\{\begin{array}{l} {\hat{x}}_{k|k}={\hat{x}}_{k|k-1}+K_{k}\left(z_{k}-{\hat{z}}_{k|k-1}\right)\\ K_{k}=P_{xz,k|k-1}{\left(P_{zz,k|k-1}\right)}^{-1}\\ P_{k|k}=P_{k|k-1}-K_{k}P_{zz,k|k-1}K_{k}^{Τ} \end{array}\right.$$

By the analyses of Equations (2)–(7), it is found that the CKF is established on the basis of a Gaussian noise system, which the SNCM $Q_{k}$ and MCNM $R_{k}$ are constant. However, the underwater system is non-Gaussian noise system, so in this case, the CKF has a low estimation accuracy.

## 3. Inverse Wishart-Based Prior Modeling of System Noise

### 3.1. Measurement Likelihood PDF

According to the Bayesian principle, the IW distribution can be seen as the conjugate prior of the unknown covariance matrix of Gaussian distribution, and the PDF of IW distribution is shown as [30]:

(8) $$\mathrm{IW}\left(B;\zeta ,\Psi \right)=\frac{|\Psi {|}^{\zeta /2}|B{|}^{-(\zeta +d+1)/2}e^{-tr(\Psi B^{-1})/2}}{2^{d\zeta /2}\Gamma_{d}\left(\zeta /2\right)}$$

where B and Ψ are the positive definite random matrix and the inverse scale matrix, respectively; tr(⋅) represents the trace operation of matrix; $\Gamma_{d}(\cdot )$ is the *d*-variate Gamma function; ζ and *d* are the degrees of freedom (DoF) parameter and the dimension of the matrix B.

The expected value of the positive random matrix B can be calculated as follows:

(9) $$B\sim \mathrm{IW}\left(\zeta ,\Psi \right)\Rightarrow E\left[B^{-1}\right]=\left(\zeta -d-1\right)\Psi^{-1}$$

Because state noise $w_{k}$ and measurement noises $v_{k}$ represent non-Gaussian noise, the IW distribution can be seen as the conjugate prior of NCM. Then, the likelihood PDFs of state transition and measurement are established.

As analyses of Equations (8) and (9), the MNCM $R_{k}$ can be modeled as follows:

(10) $$p\left(R_{k}\right)=\mathrm{IW}\left(R_{k};{\hat{u}}_{k|k-1},{\hat{U}}_{k|k-1}\right)$$

where ${\hat{u}}_{k|k-1}$ and ${\hat{U}}_{k|k-1}$ are the DoF parameter and the inverse scale matrix, respectively. And the measurement likelihood PDFs can be described as follows:

(11) $$p\left(z_{k}|x_{k}\right)=\int N\left(z_{k};h\left(x_{k}\right),R_{k}\right)\mathrm{IW}\left(R_{k};{\hat{u}}_{k|k-1},{\hat{U}}_{k|k-1}\right)dR_{k}$$

### 3.2. State Transition Likelihood PDF

In the complex underwater environment, due to various sudden interferes, the statistical characteristics of state noise are more complex than measurement noise. Therefore, the model shown in Equation (11) cannot accurately describe the system state transition process. To improve the modeling accuracy, the random mixing probability vector $\tau_{k}$ is introduced [24]. Then the conditional PDF of SNCM $Q_{k}$ is modeled as follows:

(12) $$p\left(Q_{k}|\tau_{k}\right)=\sum_{j=1}^{M}\tau_{j}\mathrm{IW}\left(Q_{k};{\hat{t}}_{j,k|k-1},{\hat{T}}_{j.k|k-1}\right),\;s.t.,\;\sum_{j=1}^{M}\tau_{j}=1$$

The random mixing probability vector $\tau_{k}$ is assumed as Dirichlet distributed, i.e.,

(13) $$p\left(\tau_{k}\right)=\mathrm{Dir}\left(\tau_{k};{\hat{\alpha }}_{k|k-1},M\right)$$

where ${\hat{\alpha }}_{k|k-1}=\left[\alpha_{1},\ldots ,\alpha_{j}\right]$ is the prior concentration parameter vector satisfying $\alpha_{j}>0$. By using the categorical distributed random vector $\lambda_{k}$, Equation (12) is modeled as hierarchical Gaussian form, the categorical distributed random vector $\lambda_{k}$ is defined as:

(14) $$\lambda =[\lambda_{1},\ldots ,\lambda_{M}],\;s.t.\;\sum_{j=1}^{M}\lambda_{j}=1$$

where the categorical element $\lambda_{j}=0$ or $\lambda_{j}=1$, the categorical vector λ is an identity vector. According to above condition, the categorical distributed random vector $\lambda_{k}$ only has M possible values, i.e., $\lambda \in {\left\{e_{j}\right\}}_{j=1}^{M}$, where $e_{j}$ is the *j*-th column of a unit matrix. To model the Equation (12) as hierarchical Gaussian form, the probability of $\lambda_{k}=e_{j}$ is defined as $\tau_{j}$, i.e.,

(15) $$\mathrm{Pr}\left(\lambda_{k}=e_{j}\right)=\tau_{j},\;\;\;\;j=1,2,\ldots ,M,\;\;\;\;s.t.\sum_{j=1}^{M}\tau_{j}=1$$

where the probability value $\tau_{j}$ is the *j*-th element of the mixing probability vector $\tau_{k}$.

Using Equation (13), the PDF of the random vector $\lambda_{k}$ can be formulated by mixing probability vector $\tau_{k}$:

(16) $$p\left(\lambda_{k}|\tau_{k}\right)=\mathrm{Cat}\left(\lambda_{k};\tau_{k},M\right)$$

By Equations (12)–(17), the PDF of SNCM $Q_{k}$ can be modeled as follows hierarchical Gaussian form:

(17) $$p(Q_{k})=\prod_{j=1}^{M}{\left[\mathrm{IW}\left(Q_{k};{\hat{t}}_{j,k|k-1}{\hat{T}}_{j,k|k-1}\right)\right]}^{\lambda_{j,k}}\mathrm{Cat}\left(\lambda_{k};\tau_{k},M\right)\times \mathrm{Dir}\left(\tau_{k};{\hat{\alpha }}_{k|k-1},M\right)$$

Then the state transition likelihood PDF is described as follows:

(18) $$\begin{array}{c} p\left(x_{k}|x_{k-1}\right)=∭N\left(x_{k};f\left(x_{k-1}\right),Q_{k}\right)\prod_{j=1}^{M}{\left[\mathrm{IW}\left(Q_{k};{\hat{t}}_{j,k|k-1},{\hat{T}}_{j,k|k-1}\right)\right]}^{\lambda_{j,k}}\mathrm{Cat}\left(\lambda_{k};\tau_{k},M\right)\\ \times \mathrm{Dir}\left(\tau_{k},{\hat{\alpha }}_{k|k-1},M\right)dQ_{k}d\lambda_{k}d\tau_{k} \end{array}$$

According to Equations (11) and (18), the hierarchical Gaussian forms of state transition and measurement transition are established. In Section 4, a novel VACKF will be derived based on VB method and cubature sampling rule.

## 4. The Proposed IW-VACKF

### 4.1. VB Method

Based on the state transition likelihood PDF Equation (18) and measurement likelihood PDF Equation (11), the optimal estimate of the joint vector $\Theta_{k}≜\left\{x_{k},x_{k-1},Q_{k},R_{k},\lambda_{k},\tau_{k}\right\}$ can be obtained by using the VB method. To use the VB method, the true posterior PDF $p\left\{\Theta_{k}|z_{1:k}\right\}$ is approximated as follows [31]:

(19) $$p\left\{\Theta_{k}|z_{1:k}\right\}=\prod_{\theta_{k}\in \Theta_{k}}q^{*}\left(\theta_{k}\right)$$

where $\theta_{k}\in \Theta_{k}$. $q^{*}\left(\theta_{k}\right)$ represents the approximate posterior PDF for each parameter $\theta_{k}$. The core of the VB method is to find a factorized approximation $q^{*}\left(\theta_{k}\right)$ that is as close as possible to the true posterior $p\left\{\Theta_{k}|z_{1:k}\right\}$. This approximation assumes that the posterior distributions of the parameters are independent.

Then, the approximate posterior PDF $q^{*}\left(\theta_{k}\right)$ can be acquired by KLD minimization as follows:

(20) $$q^{*}\left(\theta_{k}\right)=\arg\underset{q\left(\theta_{k}\right)}{\min}\mathrm{KLD}\left(\prod_{\theta_{k}\in \Theta_{k}}q\left(\theta_{k}\right)∥p\left(\Theta_{k}|z_{1:k}\right)\right)$$

where the KLD between the prior PDF and posterior PDF is calculated as follows:

(21) $$\mathrm{KLD}\left(q\left(\theta_{k}\right)||p\left(\theta_{k}\right)\right)=\int q\left(\theta_{k}\right)\log\left(\frac{q\left(\theta_{k}\right)}{p\left(\theta_{k}\right)}\right)d\theta_{k}$$

Equation (21) defines the KLD, which measures the difference between two probability distributions: the approximate posterior $q\left(\theta_{k}\right)$ and the true $p\left(\theta_{k}\right)$. Minimizing this divergence means we are seeking an approximate distribution $q^{*}\left(\theta_{k}\right)$ that best matches the true posterior $p\left\{\Theta_{k}|z_{1:k}\right\}$ under the assumption of Equation (19).

Then, the optimal approximate PDF solution can be obtained as follows:

(22) $$\log q^{*}(\theta_{k})=E_{\Theta_{k}^{\left(-\theta_{k}\right)}}^{*}\left[\log p\left(\Theta_{k},z_{1:k}\right)\right]+c_{\theta_{k}}$$

where $\Theta_{k}^{\left(-\theta_{k}\right)}$ satisfies $\left\{\theta_{k}\right\}\cup \Theta_{k}^{\left(-\theta_{k}\right)}$. Equation (22) provides the general form for updating the logarithm of the approximate posterior for a single parameter $\theta_{k}$. It states that this log-posterior is proportional to the expectation of the logarithm of the joint probability of all parameters $\theta_{k}$ and observations $z_{1:k}$, where the expectation $E_{\Theta_{k}^{\left(-\theta_{k}\right)}}^{*}$ is taken with respect to all other parameters $\Theta_{k}^{\left(-\theta_{k}\right)}$ using their current optimal approximate distributions $q^{*}\left(\cdot \right)$. The term $c_{\theta_{k}}$ is a normalization constant.

By using the fixed-point iteration method, the mutual coupling of the variational parameters can be addressed, then Equation (22) is transformed as:

(23) $$\log q^{\left(i+1\right)}\left(\theta_{k}\right)=E_{\Theta_{k}^{\left(-\theta_{k}\right)}}^{\left(i\right)}\left[\log p\left(\Theta_{k},z_{1:k}\right)\right]+c_{\theta_{k}}$$

Equation (23) specifies the iterative update rule. In the *i +* 1-th iteration, the logarithm of the approximate posterior for $\theta_{k}$, denoted $\log q^{\left(i+1\right)}(\theta_{k})$, is updated using the expectations $E_{\Theta_{k}^{\left(-\theta_{k}\right)}}^{*}$ calculated with the approximate posteriors $q^{\left(i\right)}\left(\cdot \right)$ from the previous *i*-th iteration for all parameters other than $\theta_{k}$. This iterative process continues until convergence.

Utilizing the measurement likelihood PDF Equation (11) and state transition likelihood PDF Equation (18), the joint posterior PDF is computed as:

(24) $$\begin{array}{cc} p\left(\Theta_{k},z_{1:k}\right) & =N\left(z_{k};h\left(x_{k}\right),R_{k}\right)N\left(x_{k};f\left(x_{k-1}\right),Q_{k}\right)\\ & \times \prod_{j=1}^{M}{\left[\mathrm{IW}\left(Q_{k};{\hat{t}}_{j,k|k-1},{\hat{T}}_{j,k|k-1}\right)\right]}^{\lambda_{j,k}}\mathrm{Dir}\left(\tau_{k};{\hat{\alpha }}_{k|k-1},M\right)\\ & \times \mathrm{IW}\left(R_{k};{\hat{u}}_{k|k-1},{\hat{U}}_{k|k-1}\right)\mathrm{Cat}\left(\lambda_{k};\tau_{k},M\right)p\left(z_{1:k-1}\right)\times N\left(x_{k-1};{\hat{x}}_{k-1|k-1},P_{k-1|k-1}\right) \end{array}$$

Equation (24) expresses the full joint probability of all parameters $\theta_{k}$ and the history measurement information $z_{1:k}$. It is constructed by multiplying the likelihoods of the current measurement $N\left(z_{k};h\left(x_{k}\right),R_{k}\right)$ and current state $N\left(x_{k};f\left(x_{k-1}\right),Q_{k}\right)$, with the prior distributions for the noise covariances $Q_{k}$ and $R_{k}$, and the prior for the previous state $N\left(x_{k};f\left(x_{k-1}\right),Q_{k}\right)$. The term $p\left(z_{1:k-1}\right)$ represents the evidence from previous measurements.

By introducing forgetting factor ρ∈(0,1] and tuning parameters $\pi_{k}$, the prior statistics parameters of system noise $\left\{{\hat{u}}_{k|k-1},{\hat{U}}_{k|k-1}\right\}$,${\left\{{\hat{t}}_{j,k|k-1},{\hat{T}}_{j,k|k-1}\right\}}_{j=1}^{M}$, the prior concentration parameter vector ${\hat{\alpha }}_{k|k-1}$ can be calculated as follows:

(25) $$\left\{\begin{array}{l} {\hat{\alpha }}_{k|k-1}=\rho {\hat{\alpha }}_{k-1|k-1},{\hat{t}}_{1,k|k-1}=\rho {\hat{t}}_{k-1|k-1}\\ {\hat{T}}_{1,k|k-1}=\rho {\hat{T}}_{k-1|k-1},{\hat{t}}_{j,k|k-1}=\pi_{k},{\hat{T}}_{j,k|k-1}=\pi_{k}{\hat{Q}}_{j,k}\\ {\hat{u}}_{k|k-1}=\rho {\hat{u}}_{k|k-1},{\hat{U}}_{k|k-1}=\rho {\hat{U}}_{k-1|k-1} \end{array}\right.$$

where j=2,3,…,M; ${\hat{Q}}_{j,k}$ represents the *j*-th nominal SNCM. Equation (25) give detailed explanations about the prior parameters for the noise distributions at time *k* are predicted from their posterior estimates at time *k*−1. The forgetting factor ρ allows for adapting to time-varying noise characteristics by down-weighting past information. The tuning parameters $\pi_{k}$ can provide a lower bound or ensure sufficient degrees of freedom for the Inverse-Wishart distribution of the system noise covariance.

### 4.2. Joint Inference

According to the joint posterior PDF Equation (24) and the fixed-point iteration Equation (23), the system parameters $\theta_{k}\in \Theta_{k}$ can be calculated.

Setting $\theta_{k}=x_{k}$, $\log q^{\left(i+1\right)}(\theta_{k})$ is updated as:

(26) $$\begin{array}{cc} \log q^{\left(i+1\right)}\left(\theta_{k}\right)= & -0.5{\left(z_{k}-h\left(x_{k}\right)\right)}^{Τ}E_{k}^{\left(i+1\right)}\left(R_{k}^{-1}\right)\left(z_{k}-h\left(x_{k}\right)\right)\\ & -0.5\left(x_{k}-f{\left(x_{k-1}\right)}^{T}\right)E_{k}^{\left(i+1\right)}\left(Q_{k}^{-1}\right)\times \left(x_{k}-f\left(x_{k-1}\right)\right)+c_{x_{k}} \end{array}$$

while

(27) $$q^{\left(i+1\right)}\left(x_{k}\right)=N\left(x_{k};{\hat{x}}_{k|k}^{\left(i+1\right)},P_{k|k}^{\left(i+1\right)}\right)$$

Equation (26) is derived from the general VB update rule specifically for the state $x_{k}$. It shows that the log-posterior of $x_{k}$ is a sum of quadratic terms related to the measurement prediction error $\left(z_{k}-h\left(x_{k}\right)\right)$ and the state prediction error $\left(x_{k}-f\left(x_{k-1}\right)\right)$, weighted by the expected inverse covariance matrices $E_{k}^{\left(i+1\right)}\left(R_{k}^{-1}\right)$ and $E_{k}^{\left(i+1\right)}\left(Q_{k}^{-1}\right)$ from the previous iteration. This form implies that the updated approximate posterior for $x_{k}$, $q^{\left(i+1\right)}\left(x_{k}\right)$, will be a Gaussian distribution, as shown in Equation (27), with mean ${\hat{x}}_{k|k}^{\left(i+1\right)}$ and covariance $P_{k|k}^{\left(i+1\right)}$.

Where the parameters ${\hat{x}}_{k|k}^{\left(i+1\right)}$ and ${\hat{P}}_{k|k}^{\left(i+1\right)}$ can be approximately obtained by:

(28) $$\left\{\begin{array}{l} K_{k}^{\left(i+1\right)}\approx {\tilde{Q}}_{k}^{\left(i+1\right)}\dot{h}{\left(x_{k}^{\left(i\right)}\right)}^{Τ}{\left(\dot{h}\left(x_{k}^{\left(i\right)}\right){\tilde{Q}}_{k}^{\left(i\right)}\dot{h}{\left(x_{k}^{\left(i\right)}\right)}^{Τ}+{\tilde{R}}_{k}^{\left(i\right)}\right)}^{-1}\\ {\hat{x}}_{k|k}^{\left(i+1\right)}={\hat{x}}_{k|k-1}+K_{k}^{\left(i+1\right)}\left(z_{k}-{\hat{z}}_{k|k-1}\right)\\ P_{k|k}^{\left(i+1\right)}\approx \left(I_{n}-K_{k}^{\left(i+1\right)}\dot{h}\left(x_{k}^{\left(i\right)}\right)\right){\tilde{Q}}_{k}^{\left(i+1\right)} \end{array}\right.$$

where ${\hat{x}}_{k|k-1}$ and ${\hat{z}}_{k|k-1}$ are the state prediction vector and measurement prediction vector, respectively; $\dot{h}\left(x_{k}^{\left(i\right)}\right)$ represents the first-order differentiation of a measurement process, and they can be calculated as Equations (4) and (6). The modified SNCM ${\tilde{Q}}_{k}^{\left(i\right)}$ and modified MNCM ${\tilde{R}}_{k}^{\left(i\right)}$ are calculated as follows:

(29) $${\tilde{Q}}_{k}^{\left(i\right)}={\left\{E^{\left(i\right)}\left[Q_{k}^{-1}\right]\right\}}^{-1},{\tilde{R}}_{k}^{\left(i\right)}={\left\{E^{\left(i\right)}\left[R_{k}^{-1}\right]\right\}}^{-1}$$

Based on Equations (26)–(29), setting $\theta_{k}=x_{k-1}$, $q^{\left(i+1\right)}\left(x_{k-1}\right)$ can be analytically updated as Gaussian PDF, i.e.,

(30) $$q^{\left(i+1\right)}(x_{k-1})=N\left(x_{k-1};{\hat{x}}_{k-1|k}^{\left(i+1\right)},P_{k-1|k}^{\left(i+1\right)}\right)$$

Considering that the state vector $x_{k-1}$ and prediction covariance matrix $P_{k-1}$ have been calculated at time *k*−1. Therefore, to reduce computation burden, the estimation results of the state vector $x_{k-1}$ and prediction covariance matrix $P_{k-1}$ at time *k*−1 are directly used as the optimal estimation result, i.e.,

(31) $${\hat{x}}_{k-1|k}^{*}={\hat{x}}_{k-1|k-1},{\hat{P}}_{k-1|k}^{*}=P_{k-1|k-1}$$

Similar to Equations (26)–(29), setting $\theta_{k}=R_{k}$ and $\theta_{k}=Q_{k}$, $q^{\left(i+1\right)}\left(R_{k}\right)$ and $q^{\left(i+1\right)}\left(Q_{k}\right)$ are updated as inverse Wishart PDFs:

(32) $$\left\{\begin{array}{l} q^{\left(i+1\right)}=\mathrm{IW}\left(Q_{k};{\hat{t}}_{k|k}^{\left(i+1\right)},{\hat{T}}_{k|k}^{\left(i+1\right)}\right)\\ q^{\left(i+1\right)}=\mathrm{IW}\left(R_{k};{\hat{u}}_{k|k}^{\left(i+1\right)},{\hat{U}}_{k|k}^{\left(i+1\right)}\right) \end{array}\right.$$

where the statistics parameters of system noise $\left\{{\hat{u}}_{k|k}^{\left(i+1\right)},{\hat{U}}_{k|k}^{\left(i+1\right)}\right\}$, ${\left\{{\hat{t}}_{j,k|k},{\hat{T}}_{j,k|k}\right\}}_{j=1}^{M}$ are updated as follows:

(33) $$\left\{\begin{array}{l} {\hat{t}}_{k|k}^{\left(i+1\right)}=\sum_{j=1}^{M}E^{\left(i\right)}\left[\kappa_{j,k}\right]{\hat{t}}_{j,k|k-1}+1\\ {\hat{T}}_{k|k}^{\left(i+1\right)}=\sum_{j=1}^{M}\left|E^{\left(i\right)}\kappa_{j,k}\right|{\hat{T}}_{j,k|k-1}+A_{k}^{\left(i+1\right)}\\ {\hat{u}}_{k|k}^{\left(i+1\right)}={\hat{u}}_{k|k-1}+1,\;{\hat{U}}_{k|k}^{\left(i+1\right)}={\hat{U}}_{k|k-1}+B_{k}^{\left(i+1\right)}\\ A_{k}^{\left(i+1\right)}=E^{\left(i+1\right)}\left[\left(x_{k}-f\left(x_{k-1}\right)\right){\left(x_{k}-f\left(x_{k-1}\right)\right)}^{Τ}\right]\\ B_{k}^{\left(i+1\right)}=E^{\left(i+1\right)}\left[\left(z_{k}-h\left(x_{k}\right)\right){\left(z_{k}-h\left(x_{k}\right)\right)}^{Τ}\right] \end{array}\right.$$

Equation (32) states that the posteriors for the noise covariance matrices $Q_{k}$ and $R_{k}$ are Inverse-Wishart distributions. Equation (33) shows how their parameters are updated. For $Q_{k}$, the update incorporates a sum over its *M* mixture components from the prior, weighted by the expected mixing proportions $E^{\left(i\right)}\left[\kappa_{j,k}\right]$, and adds the expected squared state prediction error $A_{k}^{\left(i+1\right)}$. For $R_{k}$, the prior parameters are updated with the expected squared measurement prediction error $B_{k}^{\left(i+1\right)}$.

According to Equations (26)–(29), setting $\theta_{k}=\lambda_{k}$, $q^{\left(i+1\right)}\left(\lambda_{k}\right)$ is renewed as categorical PMF:

(34) $$q^{\left(i+1\right)}\left(\lambda_{k}\right)=\mathrm{Cat}\left(\lambda_{k};{\hat{\beta }}_{k}^{\left(i+1\right)},M\right)$$

where ${\hat{\beta }}_{k}^{\left(i+1\right)}=\left[{\hat{\beta }}_{1,k}^{\left(i+1\right)},\ldots ,{\hat{\beta }}_{M,k}^{\left(i+1\right)}\right]$ represents the mixing probability vector estimate, and it can be determined:

(35) $$\left\{\begin{array}{l} {\hat{\beta }}_{k}^{\left(i+1\right)}=\beta_{k}^{\left(i+1\right)}/\sum_{j=1}^{M}\beta_{j,k}^{\left(i+1\right)}\\ \beta_{j,k}^{\left(i+1\right)}=\exp\left\{\begin{array}{l} E^{\left(i+1\right)}\left[\log\tau_{j,k}\right]+0.5{\hat{t}}_{j,k|k-1}\log\left|{\hat{T}}_{J,k|k-1}\right|-0.5tr\left({\hat{T}}_{j,k|k-1}E^{\left(i+1\right)}\left[Q_{k}^{-1}\right]\right)\\ -0.5\left({\hat{t}}_{j,k|k-1}+n+1\right)\times E^{\left(i+1\right)}\left[\log\left|Q_{k}\right|\right]-\log\Gamma_{n}\left(0.5{\hat{t}}_{j,k|k-1}\right)-0.5n{\hat{t}}_{j,k|k-1}\log2 \end{array}\right\} \end{array}\right.$$

where $\Gamma_{n}\left(\cdot \right)$ means the *n*-variate gamma function.

Based on Equations (26)–(29), setting $\theta_{k}=\tau_{k}$, $q^{\left(i+1\right)}\left(\tau_{k}\right)$ is modeled as Dirichlet PDF as follows:

(36) $$q^{\left(i+1\right)}\left(\tau_{k}\right)=\mathrm{Dir}\left(\tau_{k};{\hat{\alpha }}_{k|k}^{\left(i+1\right)},M\right)$$

where ${\hat{\alpha }}_{k|k}^{\left(i+1\right)}$ is the concentration parameter vector, and it can be updated by:

(37) $${\hat{\alpha }}_{k|k}^{\left(i+1\right)}={\hat{\alpha }}_{k|k-1}+E^{\left(i+1\right)}\left[\lambda_{k}\right]$$

where ${\hat{\alpha }}_{k}^{\left(i+1\right)}=\left[{\hat{\alpha }}_{1,k}^{\left(i+1\right)},\ldots ,{\hat{\alpha }}_{M,k}^{\left(i+1\right)}\right]$ is the concentration parameter vector estimate.

The SNCM $Q_{k}$ and MNCM $R_{k}$ are rewritten as inverse Wishart PDFs, so $Q_{k}^{-1}$ and $R_{k}^{-1}$ are Wishart distribution as follows:

(38) $$\left\{\begin{array}{l} q^{\left(i+1\right)}\left(Q_{k}^{-1}\right)=W\left(Q_{k}^{-1};{\hat{t}}_{k|k}^{\left(i+1\right)},{\left\{{\hat{T}}_{k|k}^{\left(i+1\right)}\right\}}^{-1}\right)\\ q^{\left(i+1\right)}\left(R_{k}^{-1}\right)=W\left(R_{k}^{-1};{\hat{t}}_{k|k}^{\left(i+1\right)},{\left\{{\hat{R}}_{k|k}^{\left(i+1\right)}\right\}}^{-1}\right) \end{array}\right.$$

By using (56), $E^{\left(i+1\right)}\left[Q_{k}^{-1}\right]$, $E^{\left(i+1\right)}\left[R_{k}^{-1}\right]$ and $E^{\left(i+1\right)}\left[\log\left|Q_{k}\right|\right]$ can be renewed as:

(39) $$\left\{\begin{array}{l} E^{\left(i+1\right)}\left[Q_{k}^{-1}\right]={\hat{t}}_{k|k}^{\left(i+1\right)}{\left\{{\hat{T}}_{k|k}^{\left(i+1\right)}\right\}}^{-1}\\ E^{\left(i+1\right)}\left[R_{k}^{-1}\right]={\hat{t}}_{k|k}^{\left(i+1\right)}{\left\{{\hat{U}}_{k|k}^{\left(i+1\right)}\right\}}^{-1}\\ E^{\left(i+1\right)}\left[\log\left|Q_{k}\right|\right]=\log\left|{\hat{T}}_{k|k}^{\left(i+1\right)}\right|-n\log2-\psi_{n}\left(0.5{\hat{t}}_{k|k}^{\left(i+1\right)}\right) \end{array}\right.$$

where $\psi_{n}(\cdot )$ denotes the n-variate digamma function.

According to the Gaussian approximations of $q^{\left(i+1\right)}\left(x_{k}\right)$ and $q^{\left(i+1\right)}\left(x_{k-1}\right)$ in Equations (27)–(31), auxiliary matrices $A_{k}^{\left(i+1\right)}$ and $B_{k}^{\left(i+1\right)}$ are calculated as:

(40) $$\left\{\begin{array}{l} A_{k}^{\left(i+1\right)}\approx \dot{f}\left(x_{k-1}\right){\hat{P}}_{k-1|k-1}^{*}\dot{f}{\left(x_{k-1}\right)}^{Τ}+P_{k|k}^{*}+\left({\hat{x}}_{k|k}^{\left(i+1\right)}-{\hat{x}}_{k|k-1}\right)\times {\left({\hat{x}}_{k|k}^{\left(i+1\right)}-{\hat{x}}_{k|k-1}\right)}^{Τ}\\ B_{k}^{\left(i+1\right)}\approx \dot{h}(x_{k}^{\left(i\right)}){\hat{P}}_{k-1|k}^{*}\dot{h}{\left(x_{k}^{\left(i\right)}\right)}^{Τ}+P_{k|k}^{*}+\left(z_{k}-{\hat{z}}_{k|k-1}\right)\times {\left(z_{k}-{\hat{z}}_{k|k-1}\right)}^{Τ} \end{array}\right.$$

where $\psi \left(\cdot \right)$ denotes the digamma function.

According to Equations (32)–(35), the expectation $E^{\left(i+1\right)}\left[\lambda_{k}\right]$,$E^{\left(i+1\right)}\left[\tau_{k}\right]$, $E^{\left(i+1\right)}\left[\log\tau_{j,k}\right]$ are updated as:

(41) $$\left\{\begin{array}{l} E^{\left(i+1\right)}\left[\lambda_{k}\right]={\hat{\beta }}_{k}^{\left(i+1\right)},E^{\left(i+1\right)}\left[\tau_{k}\right]={\hat{\delta }}_{k|k}^{\left(i+1\right)}/\sum_{j=1}^{M}{\hat{\delta }}_{j,k|k}^{\left(i+1\right)}\\ E^{\left(i+1\right)}\left[\log\lambda_{j,k}\right]=\psi \left({\hat{\delta }}_{j,k|k}^{\left(i+1\right)}\right)-\psi \left(\sum_{j=1}^{M}{\hat{\delta }}_{j,k|k}^{\left(i+1\right)}\right) \end{array}\right.$$

The pseudo code of the proposed IW-VACKF is given in Algorithm 1, where ε denotes the termination threshold and $N_{m}$ represents the upper limit of the number of iterations.
**Algorithm 1:** Time recursion of IW-VACKF**Inputs**: ${\hat{x}}_{k-1|k-1}$, $P_{k-1|k-1}$, $f\left(\cdot \right)$, $h\left(\cdot \right)$, $z_{k}$, ${\left\{{\tilde{Q}}_{j,k}\right\}}_{j=1}^{M}$, ${\hat{\alpha }}_{k-1|k-1}$, ${\hat{u}}_{k-1|k-1}$, ${\hat{U}}_{k-1|k-1}$, ${\hat{t}}_{k-1|k-1}$, ${\hat{T}}_{k-1|k-1}$, $\pi_{k}$, ρ, M, ε, $N_{m}$ **Time update** (1) Calculate ${\hat{x}}_{k|k-1}$ and ${\hat{z}}_{k|k-1}$ based on cubature sampling rule according to Equations (3)–(6) (2) Update ${\hat{\alpha }}_{k|k-1}$, ${\left\{{\hat{t}}_{j,k|k-1},{\hat{T}}_{j,k|k-1}\right\}}_{j=1}^{M}$, $\left\{{\hat{u}}_{k|k-1},{\hat{U}}_{k|k-1}\right\}$ by using Equation (25) **Measurement update**: (3) Initialization: $E^{\left(0\right)}\left[\log\tau_{j,k}\right]=\psi \left({\hat{\alpha }}_{j,k|k-1}\right)-\psi \left(\sum_{j=1}^{M}{\hat{\alpha }}_{j,k|k-1}\right)$, $E^{\left(0\right)}\left[R_{k}^{-1}\right]={\hat{u}}_{k-1|k-1}{\hat{U}}_{k-1|k-1}^{-1}$, $E^{\left(0\right)}\left[\lambda_{k}\right]={\hat{\alpha }}_{k|k-1}/\sum_{j=1}^{M}{\hat{\alpha }}_{j,k|k-1}$, ${\hat{x}}_{k-1|k}^{*}={\hat{x}}_{k-1|k-1}$, ${\hat{P}}_{k-1|k}^{*}=P_{k-1|k-1}$, $E^{\left(0\right)}\left[Q_{k}^{-1}\right]=\left(\sum_{j=1}^{M}E^{\left(0\right)}\left[\lambda_{j,k}\right]{\hat{t}}_{j,k|k-1}\right){\left(\sum_{j=1}^{M}E^{\left(0\right)}\left[\lambda_{j,k}\right]{\hat{T}}_{j,k|k-1}\right)}^{-1}$ **for**
$i=0:N_{m}-1$ (4) Calculate $x_{k|k}^{\left(i+1\right)}$ and $P_{k|k}^{\left(i+1\right)}$ by Equations (27)–(29) (5) Update $A_{k}^{\left(i+1\right)}$ and $B_{k}^{\left(i+1\right)}$ according to Equation (40) (6) Calculate ${\left\{{\hat{t}}_{j,k|k}^{\left(i+1\right)},{\hat{T}}_{j,k|k}^{\left(i+1\right)}\right\}}_{j=1}^{M}$ and $\left\{{\hat{u}}_{k|k}^{\left(i+1\right)},{\hat{U}}_{k|k}^{\left(i+1\right)}\right\}$ employing Equations (32) and (33) (7) Update $E^{\left(i+1\right)}\left[Q_{k}^{-1}\right]$, $E^{\left(i+1\right)}\left[R_{k}^{-1}\right]$, $E^{\left(i+1\right)}\left[\log\left|Q_{k}\right|\right]$ based on Equation (39) (8) Renew the mixing probability vector ${\hat{\beta }}_{k}^{\left(i+1\right)}$ by Equation (35) (9) Calculate $E^{\left(i+1\right)}\left[\lambda_{k}\right]$ by Equation (41) (10) Renew the concentration parameter vector ${\hat{\alpha }}_{k|k}^{\left(i+1\right)}$ according to Equation (37) (11) Update $E^{\left(i+1\right)}\left[\tau_{k}\right]$ and $E^{\left(i+1\right)}\left[\log\tau_{j,k}\right]$ according to Equation (41) (12) If $i\geq N_{m}$ or $\left\|{\hat{x}}_{k|k}^{\left(i+1\right)}-x_{k|k}^{\left(i\right)}\right\|/\left\|x_{k|k}^{\left(i\right)}\right\|\leq \varepsilon$, iteration is finished **end for** (13) ${\hat{x}}_{k|k}={\hat{x}}_{k|k}^{\left(i\right)}$, $P_{k|k}=P_{k|k}^{\left(i\right)}$, ${\hat{\alpha }}_{k|k}={\hat{\alpha }}_{k|k}^{\left(i\right)}$, ${\hat{u}}_{k|k}={\hat{u}}_{k|k}^{\left(i\right)}$, ${\hat{U}}_{k|k}={\hat{U}}_{k|k}^{\left(i\right)}$, ${\hat{t}}_{k|k}={\hat{t}}_{k|k}^{\left(i\right)}$, ${\hat{T}}_{k|k}={\hat{T}}_{k|k}^{\left(i\right)}$ **Outputs:**
${\hat{x}}_{k|k}$, $P_{k|k}$, ${\hat{\alpha }}_{k|k}$, ${\hat{u}}_{k|k}$, ${\hat{U}}_{k|k}$, ${\hat{t}}_{k|k}$, ${\hat{T}}_{k|k}$.

### 4.3. Discussions

**Figure 2:**
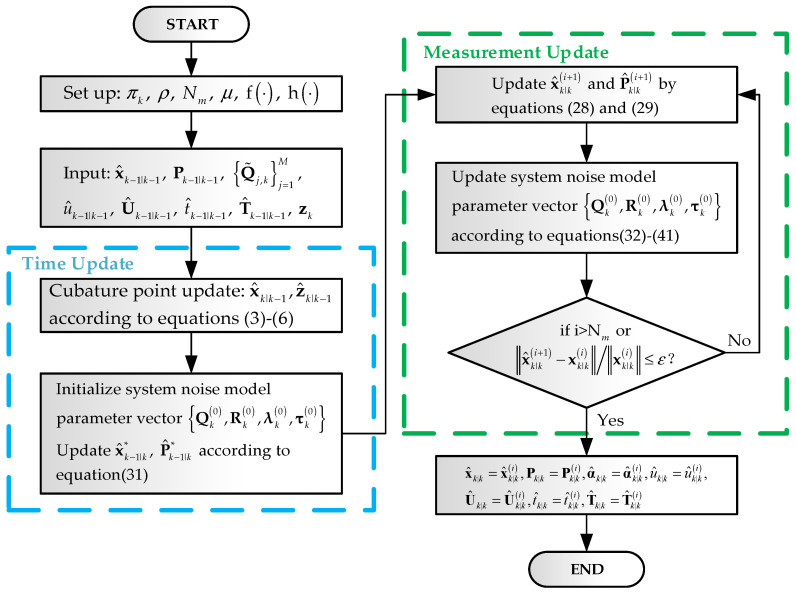
The flow chart of proposed IW-VACKF.

The proposed IW-VACKF algorithm, illustrated in Figure 2, operates two main stages: time update and measurement update. During the time update, the state one-step prediction vector and the measurement one-step prediction vector are updated using the cubature sampling rule. Critically, to account for non-stationary state and measurement noise, prior system noise parameters are updated by introducing a forgetting factor ρ and tuning parameters $\pi_{k}$. The measurement update stage renews the state estimation and prediction covariance matrix, with system noise model parameters updated via iterative calculations derived from the VB method.

(1) Parameter Selection

Reasonable parameter choice is crucial for the successful implementation of the IW-VACKF. The forgetting factor ρ in Equation (25), typically set as $\rho \in \left[0.9,1\right]$ [32]. Tuning parameters $\pi_{k}$ to adjust the DoF for the SNCM; larger values are suitable for stable state noise, while smaller values are used for unstable noise changes. The parameter M for the state noise model is set to balance the accuracy and computing burden. Initial DoF parameters for the IW distributions have an influence on the initial uncertainty of noise covariance estimates for the IW-VACKF. ${\hat{\alpha }}_{k|k-1}$ is centration parameter vector. The initial value of ${\hat{\alpha }}_{k|k-1}$ is a vector of all 1s with a length of 1 × M and update by Equation (37). To ensure the SNCM $Q_{k}^{\left(i\right)}$ and the MNCM $R_{k}^{\left(i\right)}$ converge to their true values, the initial MNCM and the initial SNCM must include the expected range of SNCM variations. To reduce computational burden, MNCM and SNCM are often set as diagonal matrices.

(2) Theoretical Advantages and Computational Complexity

Compared to existing VB adaptive filters like VBCKF-QR, the theoretical advantage of IW-VACKF lies in more accurate estimation of the SNCM and MNCM. Based on the VB structure, the IW-VACKF uses multiple IW distributions to enhance the accuracy of noise and state estimation. Regarding computational complexity, the time complexity of a standard CKF is $O\left(n^{3}\right)$, where n is the state dimension. The number of iterations is Nm. For each VB iterations, IW-VACKF updates the state estimation, so the time complexity for every step is $O\left(N_{m}\cdot M\cdot n^{3}\right)$.

## 5. Simulation and Experimental Verification

### 5.1. Simulation Test

Consider a trajectory tracking test with two fixed sensors ($S_{1}$,$S_{2}$), where $S_{1}$ is located at the origin (0,0) and $S_{2}$ is located in the position (10,10). The state vector of the target of the target is set as $x_{k}={\left[\eta_{k},\phi_{k},{\dot{\eta }}_{k},{\dot{\phi }}_{k}\right]}^{Τ}$, where $\eta_{k}$ and $\phi_{k}$ are the position coordinates of target. ${\dot{\eta }}_{k}$ and ${\dot{\phi }}_{k}$ are the corresponding velocity components. In real underwater environment, there is a pressure sensor to accurately obtain the depth information, so the simulation focuses on horizontal position information. The simulation is set [18]:

(42) $$\left\{\begin{array}{l} x_{k}=f\left(x_{k-1}\right)+w_{k}\\ z_{k}=g\left(x_{k}\right)+v_{k} \end{array}\right.$$

where $w_{k}$ and $v_{k}$ are the system noises with time-varying SNCM $Q_{k}$ and MNCM $R_{k}$, respectively, and the state transition process of the target is:

(43) $$x_{k}=\left[\begin{array}{cc} I_{2} & \Delta tI_{2}\\ 0 & I_{2} \end{array}\right]x_{k-1}+w_{k}$$

where Δt=1 s represents the sampling time; $I_{2}$ is the two-dimensional identity matrix. The SNCM at the time k is shown as follows: $Q_{k}=\left[q+0.5\cos\left(\frac{\pi k}{T}\right)\right]Q_{0}$ and $Q_{0}=\Delta \kappa \left[\begin{array}{cccc} \Delta t^{3}/3 & 0 & \Delta t^{2}/2 & 0\\ 0 & \Delta t^{3}/3 & 0 & \Delta t^{2}/2\\ \Delta t^{2}/2 & 0 & \Delta t & 0\\ 0 & \Delta t^{2}/2 & 0 & \Delta t \end{array}\right]$.

where Δκ is the scale factors selected as $\Delta \kappa =(m^{2}/m^{3})$; *q* is an adjust factor for the system state noise strength. In this simulation, by adjusting the factor *q*, the estimation consistency is verified under different state noise value. T = 150 s is the total simulation time. The measurement process is shown as follows:

(44) $$z_{k}=\left[\begin{array}{l} \sqrt{{\left(\eta_{k}-\eta_{s_{1}}\right)}^{2}+{\left(\phi_{k}-\phi_{s_{1}}\right)}^{2}}\\ \mathrm{atan}2\left(\eta_{k}-\eta_{s_{1}},\phi_{k}-\phi_{s_{1}}\right)\\ \sqrt{{\left(\eta_{k}-\eta_{s_{2}}\right)}^{2}+{\left(\phi_{k}-\phi_{s_{2}}\right)}^{2}}\\ \mathrm{atan}2\left(\eta_{k}-\eta_{s_{2}},\phi_{k}-\phi_{s_{2}}\right) \end{array}\right]+v_{k}$$

where $\left(\eta_{s_{1}},\phi_{s_{1}}\right)$ and $\left(\eta_{s_{2}},\phi_{s_{2}}\right)$ are the known position coordinates of sensors *S*_1_ and *S*_2_, respectively. The MNCMs in different sensors are selected as:  $R_{0}=\mathrm{diag}\left(10\;m^{2},0.0174\;{\mathrm{rad}}^{2}\right)$, $R_{k,1}=0.5R_{0}\left[0.1+0.05\cos\left(\frac{\pi k}{\Delta t}\right)\right]$, $R_{k,2}=0.3R_{0}\left[0.1+0.05\cos\left(\frac{\pi k}{\Delta t}\right)\right]$. The initial state is set as $x_{0}={\left[40,50,8,8\right]}^{Τ}$, and the initial prediction error covariance matrix is chosen as $P_{0|0}=\mathrm{diag}\left(\left[4,2,2,2\right]\right)$.

In the proposed IW-VACKF, the number of mixture terms is set as M=4; the initial DoF parameters are chosen as ${\hat{t}}_{0|0}=5$ and ${\hat{u}}_{0|0}=10$; the initial concentration parameter vector is selected as ${\hat{\alpha }}_{0|0}=1_{1\times 4}$; the tuning parameter $\pi_{k}$ and the forgetting factor ρ are respectively chosen as $\pi_{k}=5$, ρ=0.996. To control the number of iterations calculation, set ε and $N_{m}$ as $10^{-10}$ and 50, respectively. And the CKF with TNCM (CKF-TNCM) is set as the reference. In the CKF-TNCM, the TNCM are employed to achieve recursive state estimates.

The root-mean-square errors (RMSEs) and the average RMSEs (ARMSEs) are used as performance index to compare the state estimate accuracy in different algorithms, and they are calculated as [33]:

(45) $$\left\{\begin{array}{l} {\mathrm{RMSE}}_{pos}≜\sqrt{\frac{1}{M}\sum_{s=1}^{M}\left({\left(\eta_{k}^{s}-{\hat{\eta }}_{k}^{s}\right)}^{2}+{\left(\phi_{k}^{s}-{\hat{\phi }}_{k}^{s}\right)}^{2}\right)}\\ {\mathrm{ARMSE}}_{pos}≜\sqrt{\frac{1}{MT}\sum_{k=1}^{T}\sum_{s=1}^{M}\left({\left(\eta_{k}^{s}-{\hat{\eta }}_{k}^{s}\right)}^{2}+{\left(\phi_{k}^{s}-{\hat{\phi }}_{k}^{s}\right)}^{2}\right)} \end{array}\right.$$

where $\left(\alpha_{k}^{s},\beta_{k}^{s}\right)$ is the true position at the *s*-th Monte Carlo run, $\left({\hat{\alpha }}_{k}^{s},{\hat{\beta }}_{k}^{s}\right)$ represents the estimated positions, and the total number of Monte Carlo runs is denoted by *M*. Equation (45) shows the calculation process of the position errors. Similarly, the velocity errors can be acquired in the same way as Equation (45).

In addition, the normalized estimation errors squared (NEES) and the average normalized estimation errors squared (ANEES) are employed to evaluate the estimation consistency, and then define them as follows [34]:

(46) $$\mathrm{ANEES}=\frac{1}{MT}\sum_{k=1}^{T}\sum_{s=1}^{M}{\left(x_{k}^{s}-{\hat{x}}_{k|k}^{s}\right)}^{Τ}{\left(P_{k|k}^{s}\right)}^{-1}(x_{k}^{s}-{\hat{x}}_{k|k}^{s})$$

where $x_{k}^{s}$ and ${\hat{x}}_{k|k}^{s}$ are respectively the true and estimated system state vector at the time *k*; $P_{k|k}^{s}$ is the system estimation error covariance matrix in the *s*-th Monte Carlo run, and *M* is the total number of Monte Carlos runs. Based on the principle of state determination, if the values of ANEES are within the expected bounds around the state dimension n. It means that the theoretical estimation error covariance is consistent with the actual estimation error.

**Figure 3:**
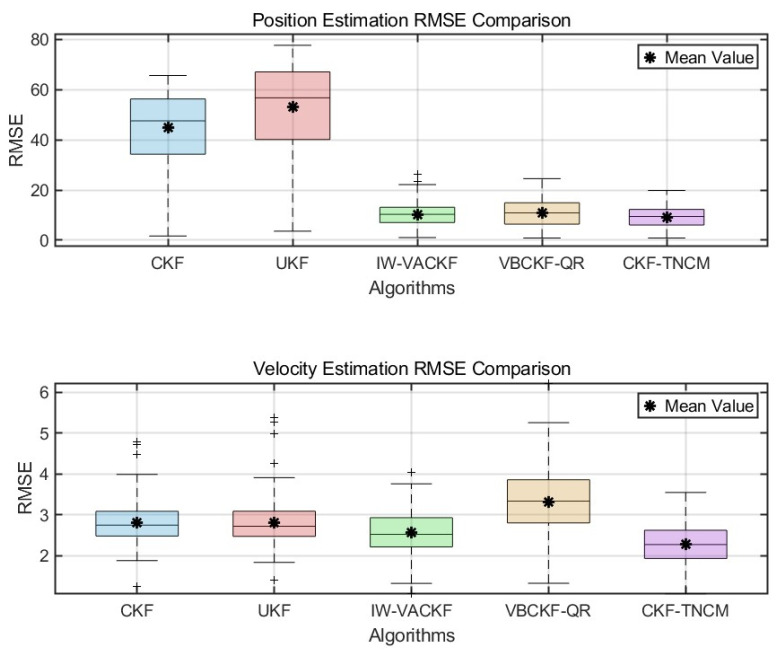
RMSE Distribution for Position and Velocity Estimation via Boxplots (*q* = 1).

To provide detailed statistical analysis and better quantify performance gains as requested, we conduct Monte Carlo simulations for the q = 1 scenario. The RMSE distributions for position and velocity are presented in the boxplot of Figure 3. Figure 3 include the median, mean, interquartile range (IQR), and outliers for each algorithm. These results demonstrate the clear advantage of the proposed IW-VACKF: its mean and median position RMSE and velocity RMSE are substantially lower than those of standard UKF and CKF. Furthermore, IW-VACKF generally exhibits a more compact RMSE distribution, indicating more consistent performance. Compared to VBCKF-QR, IW-VACKF shows comparable or slightly superior RMSE with a similarly tight distribution. This statistical evidence robustly supports the claim that IW-VACKF offers enhanced estimation accuracy.

**Figure 4:**
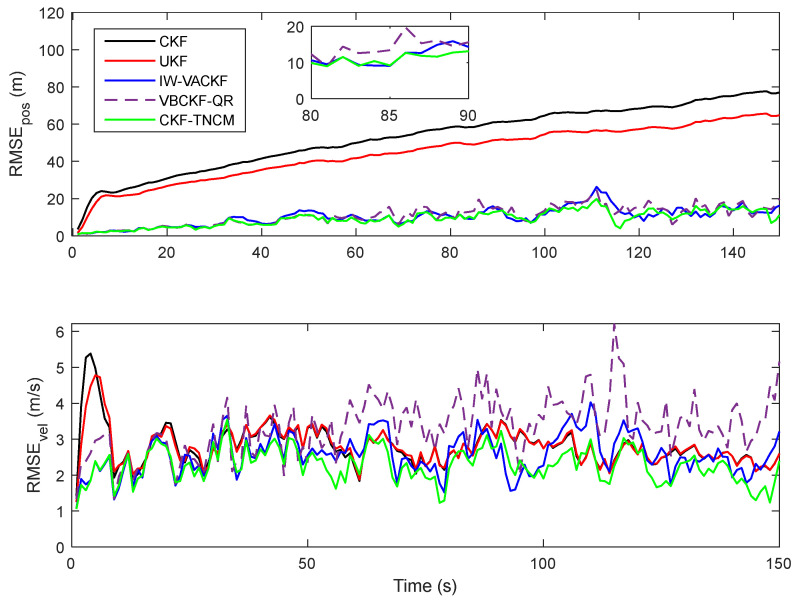
RMSE of position and velocity.

Figure 4 shows the estimate error in different filters when the adjust factor is selected as *q* = 2, the initial nominal SNCM set ${\left\{{\tilde{Q}}_{j,0}\right\}}_{j=1}^{M}$ is chosen as ${\tilde{Q}}_{1,0}=1.8I_{4}$, ${\tilde{Q}}_{2,0}=2I_{4}$, ${\tilde{Q}}_{3,0}=2.2I_{4}$ and ${\tilde{Q}}_{4,0}=2.5I_{4}$. The proposed IW-VACKF has a better performance than the compared CKF, UKF and VBCKF-QR. Table 1 shows the average state estimation accuracy under different system state noise value parameters *q*. This is a quantitative assessment of how each filter’s accuracy responds to increasing levels of uncertainty in the system model. The proposed IW-VACKF has improved position estimation precision by at least 10%.

**Figure 5:**
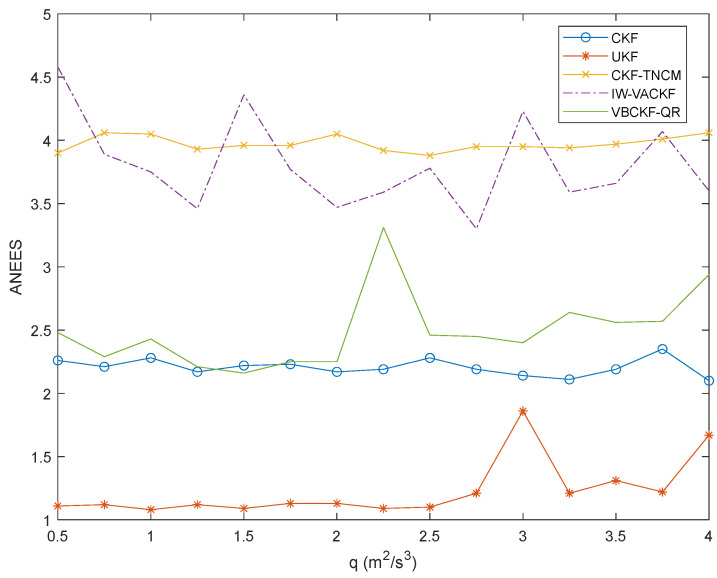
ANEES of different filter in multi-sensor tracking.

It is illustrated from Figure 5 that the IW-VACKF has the best determination consistency than the compared UKF, CKF and VBCKF-QR under different adjusting factor q. Because the SNCM and MNCM are time-vary and unknown, the existing algorithms with fixed NCMs perform poor in estimation consistency.

It is illustrated from Figure 5 that the IW-VACKF has the best determination consistency than the compared UKF, CKF and VBCKF-QR under different adjusting factor q. The proposed IW-VACKF consistently maintains its ANEES at a low level across this range, generally performing closest to the optimal CKF-TNCM which utilizes true noise covariance matrices. This indicates robust of IW-VACKF even as the system noise characteristics change. Because the SNCM and MNCM are time-vary and unknown, the existing algorithms with fixed NCMs perform poor in estimation consistency. For example, both UKF and CKF exhibit generally higher and more fluctuating ANEES values compared to IW-VACKF, while VBCKF-QR also shows less stable consistency with ANEES values that tend to be higher than those of IW-VACKF, especially for larger *q* values.

### 5.2. Experimental Verification on Underwater Vehicle

**Figure 6:**
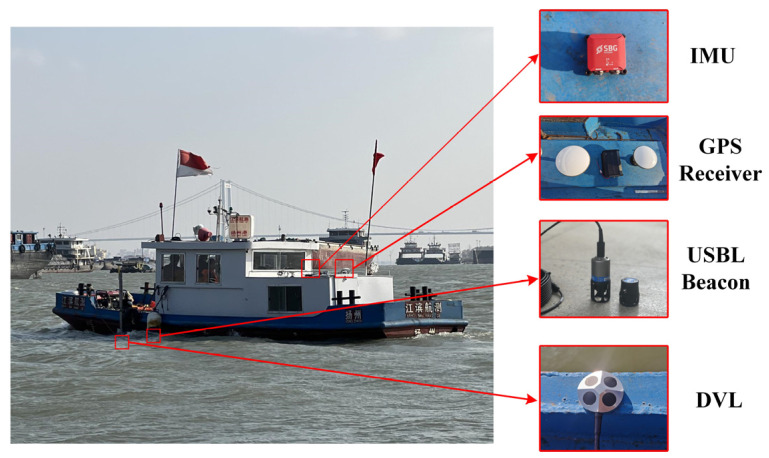
Experimental verification of ship with sensors.

**Figure 7:**
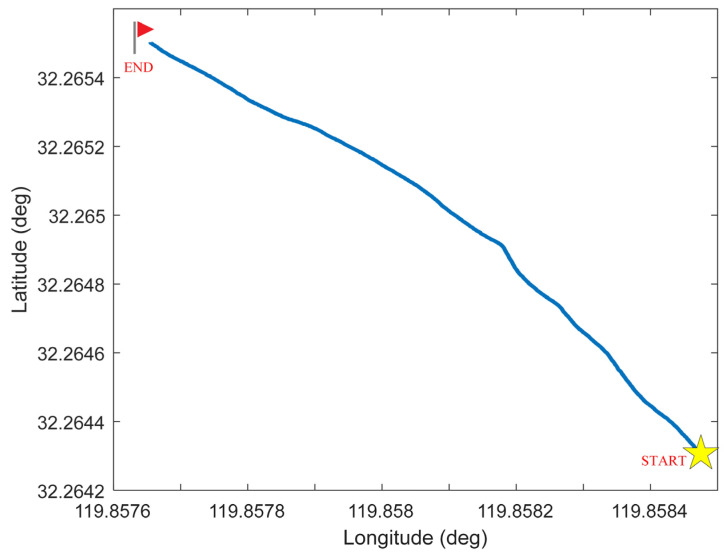
Experiment vessel trajectory.

To validate the performance of the IW-VACKF, the real experiment is performed in Yangtze River. USBL is used underwater to provide positioning information for underwater vehicle as reference, but the USBL is prone to be interfered by unknown and random noises. To evaluate the performance of the proposed method compared with other traditional methods, we develop the surface vessel experimental platform shown in Figure 6, which acquires high-precision reference data through GPS. DVL and USBL are deployed underwater to collect underwater navigation data and fusion with IMU. Detailed specifications of the IMU and DVL are provided in Table 2, while the reference trajectory used in the trials is shown in Figure 7.

Considering the characteristics of complex underwater noise, the IW-VACKF introduces the mixed probability vector $\tau_{k}$ on the basis of IW distribution. Then, the system can obtain an accurate model of state noise and based on the method of fixed-point iteration, the proposed IW-VACKF obtains higher precision position estimations than other compared methods.

**Figure 8:**
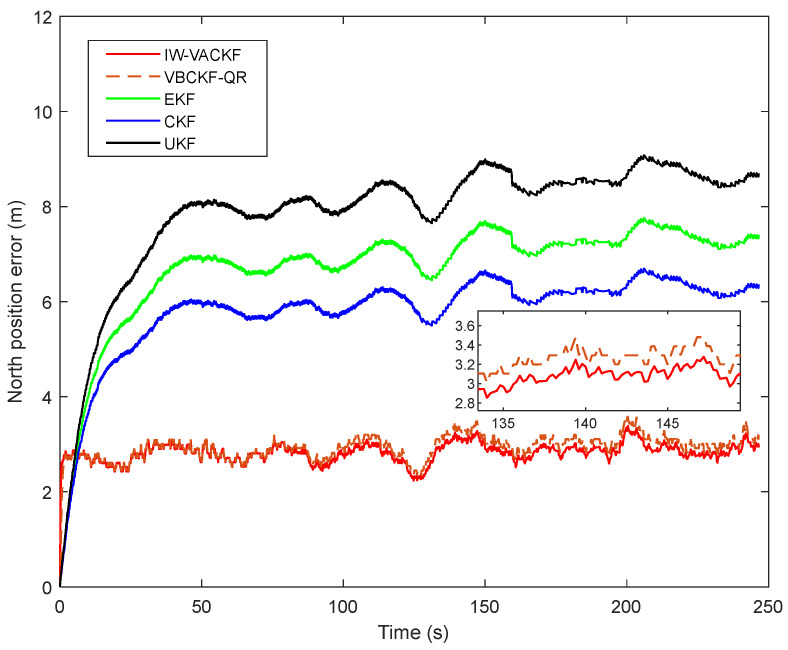
The state estimation of north direction in different algorithms.

**Figure 9:**
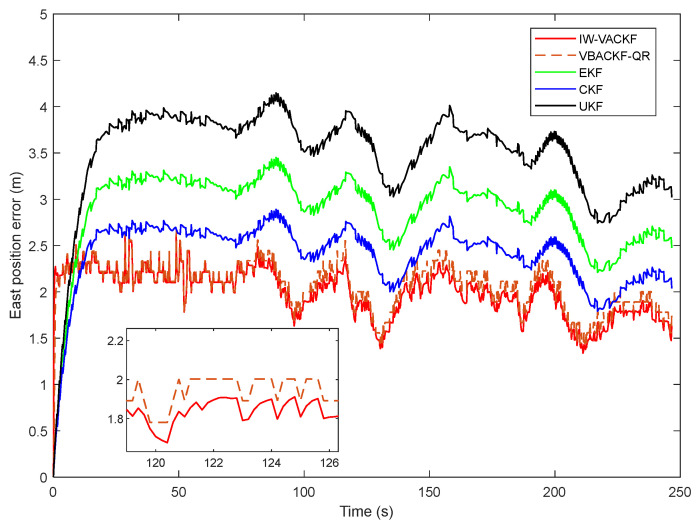
The state estimation of east direction in different algorithms.

**Figure 10:**
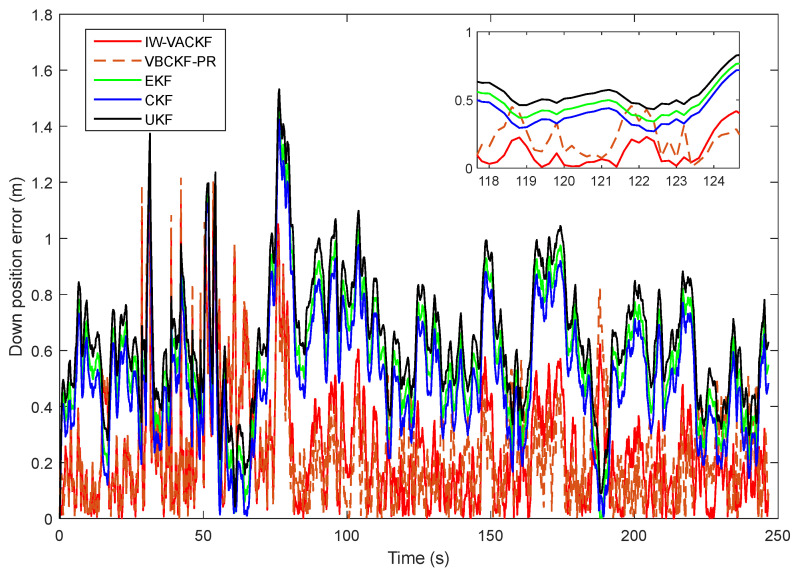
The state estimation of down direction in different algorithms.

**Figure 11:**
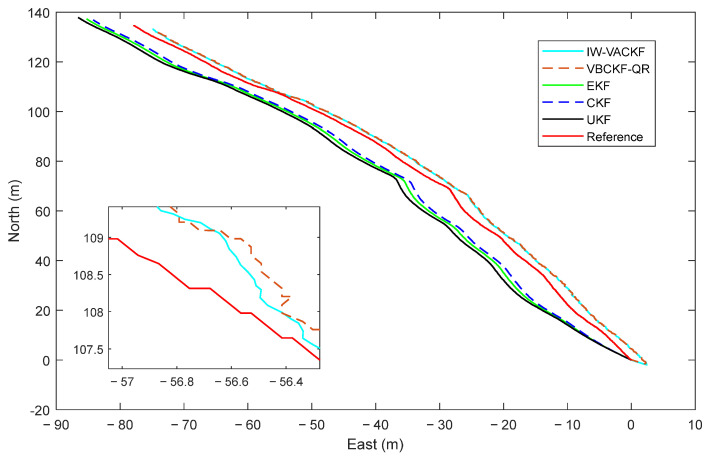
The state estimation trajectory in different algorithm.

Figure 8, Figure 9 and Figure 10 show the position determination error in different algorithms, the proposed IW-VACKF has the best performance than exiting algorithms. According to Table 3, the proposed IW-VACKF has improved accuracy by at least 4.3% than compared algorithms in north direction. In east direction, the proposed IW-VACKF has improved accuracy by at least 4.0%. And in down direction, the proposed IW-VACKF has improved accuracy by at least 4.3%. Figure 11 shows the estimated trajectories of different algorithms. As can be seen from Figure 11, the proposed method is the closest to the true value compared to other compared methods.

Validated through simulation and field tests, the proposed system model and navigation method exhibit superior performance, enabling underwater vehicles to achieve high-precision navigation and positioning.

## 6. Conclusions

This paper proposes a novel inverse-Wishart-based variational Bayesian adaptive Kalman filter algorithm, to address the complex noise problem of underwater integrated navigation systems, thereby enhancing the accuracy and robustness of multi-sensor data fusion in complex underwater environments. Firstly, based on the characteristics of underwater complex noise, the nonlinear system state transition and measurement process are described as hierarchical Gaussian model. Then, in the basis of the hierarchical Gaussian model, the system state is estimated by VB approach. Finally, by utilizing the cubature rule, the proposed IW-VACKF can be implemented using as fixed-point iteration method. Simulations and real trials demonstrate that the proposed IW-VACKF has better state estimation precision.

## Figures and Tables

**Table 1:** ARMSEs of position and velocity in multi-sensor tracking.

Parameters	Filter	${\mathrm{ARMSE}}_{pos}$ (m)	${\mathrm{ARMSE}}_{vel}$ (m/s)
q=1	UKF	53.3109	2.9186
	CKF	45.2893	2.8961
	VBCKF-QR	10.8589	3.3216
	IW-VACKF	10.2683	2.5733
	CKF-TNCM	8.9651	2.2954
q=2	UKF	61.9777	4.3249
	CKF	58.6286	4.3189
	VBCKF-QR	12.3005	3.6468
	IW-VACKF	11.2230	3.3291
	CKF-TNCM	10.6104	3.0313
q=3	UKF	73.9291	5.5212
	CKF	96.2329	7.2268
	VBCKF-QR	13.8656	4.2379
	IW-VACKF	11.4812	3.1558
	CKF-TNCM	11.4189	3.4854

**Table 2:** Parameters of sensors in surface vessel trial.

**Items**	**Gyroscope**
In run bias stability (°/h)	8
Scale factor stability (ppm)	500
Angular Random Walk $\left({}^{\circ}/\sqrt{h}\right)$	0.18
One year bias stability (°/s)	0.4
**Items**	**Accelerometers**
Scale factor stability (ppm)	1000
Velocity Random walk $\left(\mu g/\sqrt{\mathrm{Hz}}\right)$	57
In run bias instability (μg)	14
One year bias stability (mg)	5
**Items**	**GPS**
Single positional Accuracy (m)	5
Velocity Accuracy (m/s)	3
**Items**	**DVL**
Velocity Accuracy (m/s)	0.001

**Table 3:** Estimated Error of position target tracking of surface vessel.

Algorithms	North Error (m)	East Error (m)	Down Error (m)
IW-VACKF	2.82	1.99	0.23
VBCKF-QR	2.94	2.07	0.24
EKF	6.73	2.89	0.57
UKF	7.87	3.49	0.65
CKF	5.80	2.41	0.51

## Data Availability

The raw data supporting the conclusions of this article will be made available by the authors on request.
